# Effectiveness, nephrotoxicity, and therapeutic drug monitoring of polymyxin B in nosocomial pneumonia among critically ill patients

**DOI:** 10.1111/crj.13493

**Published:** 2022-05-19

**Authors:** Qinghua Ye, Qianlin Wang, Ziying Chen, Wenqian Chen, Qingyuan Zhan, Chen Wang

**Affiliations:** ^1^ Peking University China‐Japan Friendship School of Clinical Medicine Beijing China; ^2^ Department of Pulmonary and Critical Care Medicine, Center of Respiratory Medicine China‐Japan Friendship Hospital, National Clinical Research Center for Respiratory Diseases Beijing China; ^3^ Graduate School of Peking Union Medical College Chinese Academy of Medical Sciences, Peking Union Medical College Beijing China; ^4^ Department of Pharmacy China‐Japan Friendship Hospital Beijing China; ^5^ Chinese Academy of Medical Sciences and Peking Union Medical College Beijing China

**Keywords:** AUC_ss,24h_, effectiveness, nephrotoxicity, polymyxin B, therapeutic drug monitoring

## Abstract

**Objectives:**

We aimed to assess the effectiveness and nephrotoxicity of polymyxin B in critically ill patients with nosocomial pneumonia and to evaluate the utility of its therapeutic drug monitoring (TDM).

**Methods:**

We retrospectively analyzed patients who received polymyxin B treatment for ≥48 h since the establishment of polymyxin B TDM in a 26‐bed tertiary referral intensive care unit. Univariate and multivariate analyses were conducted to assess the variables associated with polymyxin B effectiveness and nephrotoxicity.

**Results:**

A total of 62 patients were enrolled. Most (56.5%) of the patients performed TDM, 54.3% of them reached the therapeutic target of area under curve across 24 h at steady state (AUC_ss,24h_) of 50–100 mg h L^−1^, and 10 patients had an AUC_ss,24h_ value of <50 mg h L^−1^. Thirty‐six (58.1) and 31 (50.0%) patients had favorable clinical and microbiological responses, respectively. Reaching the therapeutic target of AUC_ss,24h_ (odds ratio [OR] = 13.15, *p* = 0.015), a favorable microbiological response (OR = 40.80, *p* = 0.00), and complicated with septic shock (OR = 0.12, *p* = 0.021) were independently associated with favorable clinical outcomes of polymyxin B treatment. The incidence of acute kidney injury (AKI) was 45.2%. A lower creatinine clearance (OR = 0.96, *p* = 0.008) and concomitant treatment with loop diuretics (OR = 5.93, *p* = 0.046) were predictive of nephrotoxicity.

**Conclusion:**

Our findings show that TDM of polymyxin B is a valuable intervention, and the achievement of its optimal pharmacodynamic target can improve treatment outcome. Renal insufficiency and concomitant treatment with loop diuretics were found to be associated with the risk of nephrotoxicity.

## Graphical abstract

Our study evaluated the effectiveness and nephrotoxicity of polymyxin B in critically ill patients with nosocomial pneumonia and reported the utility of polymyxin B TDM for the first time. Our results show that the achievement of the optimal pharmacodynamic target for polymyxin B can improve the treatment outcomes, and renal insufficiency and concomitant treatment with loop diuretics are associated with the risk of nephrotoxicity.

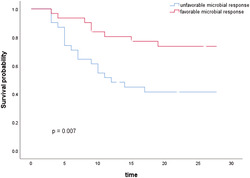

## 1 INTRODUCTION

The burgeoning antibiotic resistance has represented a formidable threat to the clinical management of nosocomial pneumonia caused by multidrug‐resistant (MDR) and extensive drug‐resistant (XDR) Gram‐negative bacilli.^1^ Due to the scarcity of new effective antibiotics, the classic polymyxins antibiotics, which remain active against these organisms, have reemerged as a last resort treatment option against these challenging infections.^2^

There are two clinically used polymyxins: colistin and polymyxin B.^3^ Parenteral polymyxin B has several advantages over colistin.^4^ It is administered directly in its active form, and therefore, intravenous administration can rapidly and reliably achieve a desired concentration. It is excreted by non‐renal clearance, meaning that dose adjustment in patients with renal dysfunction is not generally needed. Recently, published systematic reviews and meta‐analyses showed that although there is no significant difference in mortality between patients treated with these two polymyxins, polymyxin B appears to have lower nephrotoxicity.^5^, ^6^ For these reasons, polymyxin B has become the predominant polymyxin used in many centers. A number of studies have demonstrated that polymyxin B has acceptable efficacy and safety in the treatment of MDR and XDR Gram‐negative bacteria infections.^7^, ^8^, ^9^ However, scant information on its clinical application in critically ill patients with nosocomial pneumonia is available.^10^ In addition, some in vitro studies showed that polymyxin B was less effective against lung infections.^11^ Therefore, the effectiveness and nephrotoxicity of polymyxin B in this patient population warrant further investigation.

The optimization of antibiotics use in critically ill patients is recognized to be a challenging and complicated process. For this patient population, rapidly dynamic physiology and required interventions will significantly influence the pharmacokinetics (PK) that affects antibiotic exposure.^12^ Given the strong relationship between appropriate antibiotic treatment in severe infected patients and mortality, therapeutic drug monitoring (TDM) may be of value to optimize plasma concentrations of antimicrobials and thereby improve clinical outcomes.^13^ The therapeutic window of polymyxin B is narrow and almost overlaps with the threshold concentration of nephrotoxicity.^10^ In addition, polymyxins are vulnerable to induce drug resistance, and adequate drug concentration in the early stage of treatment is an important factor affecting the success of clinical treatment.^10^ Based on these characteristics, it is necessary to perform TDM during polymyxin B therapy. However, to our knowledge, no relevant studies about the introduction of polymyxin B TDM into clinical practice were reported.

Therefore, we conducted this retrospective study to assess the effectiveness and nephrotoxicity of polymyxin B in critically ill patients with nosocomial pneumonia and to evaluate the utility of its TDM.

## 2 METHODS

### 2.1 Study design

This retrospective, single‐center cohort study was conducted in the respiratory intensive care unit of the China‐Japan Friendship Hospital, National Clinical Research Center for Respiratory Diseases, a 1600‐bed teaching hospital in Beijing. The TDM of polymyxin B has been a routine item in the pharmacy department of our hospital since May 2019. All adult patients (≥18 years old) who had received intravenous polymyxin B for ≥48 h due to hospital‐acquired pneumonia (HAP) or ventilator‐associated pneumonia (VAP) between May 2019 and January 2021 were enrolled. The diagnoses of MDR or XDR HAP and VAP were confirmed by two ICU physicians following the 2016 clinical practice guidelines of the Infectious Diseases Society of America and the American Thoracic Society.^14^ Patients with severe renal dysfunction (baseline serum creatinine > 1.5 mg dl^−1^ or requiring renal replacement therapy before polymyxin B therapy) were excluded. For patients who received more than one treatment with polymyxin B, we analyzed only the first course. This study was approved and supervised by the Ethics Committee of China‐Japan Friendship Hospital (No. 2019‐156‐K105), and written informed consent was waived.

Clinical information including patient demographics characteristics, comorbidities, sequential organ failure assessment (SOFA) score, acute physiology and chronic health evaluation (APACHE) II score, ICU admission diagnosis, pertinent laboratory findings, methods of respiratory support, status of volume management, and concomitant drugs were extracted from the electronic patient medical records.

### 2.2 Variables and definitions

The Charlson comorbidity index (CCI)^15^ was used to evaluate patient comorbidities. Baseline serum creatinine was that measured on the day that polymyxin B was initiated. The RIFLE (risk, injury, failure, loss, end‐stage kidney disease) criteria,^16^ which are based on the highest serum creatinine value observed during polymyxin B therapy and compared with the baseline, were used to evaluate whether the patients had developed renal injury and the severity of nephrotoxicity. Creatinine clearance (CL_CR_) was estimated from serum creatinine levels for each patient using the Cockcroft–Gault equation.

The dose regimen and duration of polymyxin B therapy were individualized for each patient at the discretion of the attending physician; these decisions were based on the clinical context and guidelines adopted by our unit. Each dose of polymyxin B (polymyxin B sulfate for injection; 1 mg containing approximately 10 000 U) was administered as a 50‐ml infusion over a 1‐h period. The method used in the previously published literature of our team was used to determine the plasma concentration of polymyxin B and estimate the area under the concentration–time curve across 24 h at steady state (AUC_ss,24h_).^17^ For patients with TDM data, we collected the first AUC_ss,24h_ value during the treatment course. According to the guideline for optimal use of polymyxins,^10^ the target value of AUC_ss,24h_ was set to 50–100 mg h L^−1^.

Clinical efficacy was assessed according clinical and microbiological criteria after the last dose of polymyxin B. The clinical response to polymyxin B treatment was determined by comparing the patient's baseline signs and symptoms of infection with those after treatment. Favorable clinical response was defined as the significant resolution of signs and symptoms of the infection by the end of polymyxin B therapy, improvement or no worsening in procalcitonin (PCT), PaO_2_/FiO_2_ ratio (PFR), and bedside chest X‐ray. Unfavorable clinical response was defined as the occurrence of any one or more of the following circumstances: a lack of response that required additional intervention and/or other antibacterial therapy, initial recovery followed by condition deterioration before the assessment, or death during the polymyxin B therapy due to severe pneumonia. Baseline pathogens were collected from the respiration samples 5 days before to 3 days after polymyxin B therapy, and antibiotic susceptibility of microorganisms was conducted by using a BD Phoenix‐100 automated microbiology system. A favorable microbiological response was defined as the eradication of responsible pathogens from the posttreatment respiratory track cultures. If the clinical response was determined as a success and no material was available for culture, the causative pathogen(s) was presumed to be cleared. The case report form for this study was prepared by a third person and then handed over to the two physicians who made the clinical assessment. The two physicians, who were not the physicians caring for the patient at the time, would conduct independent retrospective evaluations based on the collected data. Furthermore, they were blind to the microbiological outcome, which would be evaluated by other physicians.

### 2.3 Statistical analysis

Statistical analysis was conducted using SPSS version 25.0. Categorical data are presented as frequencies (%), and continuous data are presented as the means ± standard deviations (SD) or medians (interquartile range, IQR). Student's *t* tests or Mann–Whitney *U* tests were used to analyze continuous variables between groups, and categorical variables were analyzed by the *χ* ^2^ test or Fisher's exact test. A two‐tailed *p* value < 0.05 was considered statistically significant. Logistic regression analyses were performed after the univariate analysis, in which variables that were considered clinically relevant and statistically significant were used as covariates. For the time‐to‐event analyses, Kaplan–Meier curves were used. The Cox proportional‐hazards model was used to analyze the association between risk factors and the onset of acute kidney injury (AKI).

## 3 RESULTS

A total of 62 patients were enrolled in the analysis. Key demographic and clinical characteristics are summarized in Table 1. The most common recovered bacterium was *Acinetobacter baumannii* (50.0%), followed by *Pseudomonas aeruginosa* (48.4%) and *Klebsiella pneumoniae* (17.7%). Most of isolates were found to be highly susceptible to polymyxin B by microbroth dilution testing (MIC ≤ 0.5 mg L^−1^) (Table 1). TDM was performed for 56.5% of the patients, of whom 54.3% of patients reached the target AUC_ss,24h_ value, and the rate of AUC_ss,24h_ > 100 mg h L^−1^ was 17.1% (Tables 1, 2). Ten patients (28.6%) had an AUC_ss,24h_ value of <50 mg h L^−1^, of whom five patients subsequently increased the dose of polymyxin B and seven patients responded well to polymyxin B treatment. We also compared the doses of the groups with AUC_ss,24h_ < 50 mg h L^−1^ and those reached the target value and found that there was no significant difference (1.7 ± 0.4 vs. 1.6 ± 0.3 mg kg day^−1^, *p* = 0.654). The favorable clinical response rate was 58.1%, and the microbiological eradication rate was 50.0% (Table 1). The mortality of all patients was 48.4%, with 19.5% and 88.5%, respectively, for the favorable group and unfavorable group.

**TABLE 1:** Characteristics of patients and univariate analysis for clinical response to polymyxin B

Variable	Entire cohort	Favorable response	Unfavorable response	
	*n* = 62	*n* = 36	*n* = 26	*p* value
Demographics				
Age (years)	65.2 ± 13.8	64.8 ± 16.3	65.8 ± 9.8	0.751
Female	28 (45.2)	21 (58.3)	7 (26.9)	0.013
Weight (kg)	60.7 ± 14.9	60.1 ± 16.7	61.6 ± 12.2	0.717
BMI (kg m^−2^)	22.1 ± 5.6	22.4 ± 6.0	21.8 ± 5.1	0.703
APACHE II	24.2 ± 7.1	23.1 ± 7.0	25.8 ± 7.3	0.156
SOFA	8 (5.0–11.0)	8 (5.0–10.3)	8 (4.3–13.0)	0.480
Comorbidities				
CCI score	5.1 ± 2.3	5.1 ± 2.6	5.0 ± 1.9	0.905
Chronic pulmonary disease	38 (61.3)	20 (55.6)	18 (69.2)	0.275
Chronic heart failure	21 (33.9)	11 (30.6)	10 (38.5)	0.516
Malignant disease	7 (11.3)	5 (13.9)	2 (7.7)	0.689
Diabetes	25 (40.3)	16 (44.5)	9 (34.6)	0.436
ARDS	8 (12.9)	3 (8.3)	5 (19.2)	0.262
Septic shock	28 (45.2)	12 (33.3)	16 (61.5)	0.028
Bloodstream infection	10 (16.1)	7 (19.4)	3 (11.5)	0.499
Baseline condition				
ALT (U L^−1^)	24 (13.8–42.8)	25.5 (15.0–44.3)	22.5 (10.8–43.0)	0.521
AST (U L^−1^)	35 (21.8–53.3)	36.5 (23.5–56.3)	32.5 (19.5–50.5)	0.288
ALB (g L^−1^)	35.3 ± 5.6	36.0 ± 6.2	34.3 ± 4.7	0.247
TBIL (μmol L^−1^)	12.3 (6.9–24.4)	12.3 (6.9–24.2)	12.6 (6.8–27.4)	0.875
Serum creatinine (μmol L^−1^)	70.0 ± 29.0	67.0 ± 28.0	74.0 ± 30.5	0.358
WBC (×10^9^ L^−1^)	13.8 ± 6.8	13.9 ± 7.7	13.7 ± 5.5	0.792
PCT (ng ml^−1^)	0.5 (0.2–1.8)	0.5 (0.2–1.8)	0.5 (0.2–1.5)	0.764
PFR (mmHg)	138.8 (93–222.3)	151.9 (107.3–243.2)	126.9 (84.8–160.6)	0.049
Respiratory support				
Endotracheal intubation	54 (87.1)	32 (88.9)	22 (84.6)	0.710
Tracheotomy	30 (48.4)	17 (47.2)	13 (50.0)	0.829
Pathogens and susceptibility				
*Pseudomonas aeruginosa*	30 (48.4)	18 (50.0)	12 (46.2)	0.765
*Acinetobacter baumannii*	31 (50.0)	18 (50.0)	13 (50.0)	1.00
*Klebsiella pneumoniae*	11 (17.7)	4 (11.1)	7 (26.9)	0.177
MIC ≤ 0.5 mg L^−1^	43 (69.4)	28 (77.8)	15 (57.7)	0.090
Favorable microbiological efficacy	31 (50)	27 (75.0)	4 (15.4)	0.000
Polymyxin B dose regimen				
Load at first dose	49 (79.0)	31 (86.1)	18 (69.2)	0.107
Daily dose (mg kg^−1^)	1.7 ± 0.4	1.8 ± 0.4	1.7 ± 0.3	0.581
Cumulative dose (mg)	1150 (637.5–1675.0)	1225 (712.5–1787.5)	875 (500–1450)	0.06
Treatment time (days)	11 (6–17.3)	12.5 (6.3–18)	8.5 (5–14)	0.064
TDM	35 (56.5)	24 (66.7)	11 (42.3)	0.056
AUC_SS,24h_ 50–100 mg h L^−1^	19 (54.3)	15 (62.5)	4 (36.4)	0.027
Outcomes				
AKI	28 (45.2)	17 (47.2)	11 (42.3)	0.701
Time of AKI onset (days)	6 (3–10.8)	6 (4–12)	5 (3–6)	0.226
CRRT	11 (17.7)	4 (11.1)	7 (26.9)	0.177

*Note*: Data are presented as *n* (%), mean + SD or median (IQR).

Abbreviations: AKI, acute kidney injury; ALB, albumin; ALT, alanine aminotransferase; APACHE, acute physiology and chronic health evaluation; ARDS, acute respiratory distress syndrome; AST, aspartate aminotransferase; AUC_SS,24h_, area under the concentration–time curve across 24 h at steady state; BMI, body mass index; CCI, Charlson comorbidity index; CRRT, continuous renal replacement therapy; MIC, minimum inhibitory concentration; PCT, procalcitonin; PFR, PaO_2_/FiO_2_ ratio; SOFA, sequential organ failure assessment; TBIL, total bilirubin in serum; TDM, therapeutic drug monitoring; WBC, white blood cell.

**TABLE 2:** Univariate analysis for AKI

Variables	AKI	No AKI	*p*
	*n* = 28	*n* = 34	
Demographics			
Age (years)	67.6 ± 11.5	63.2 ± 15.3	0.188
Female	12 (42.9)	16 (47.1)	0.741
Weight (kg)	62.0 ± 12.3	60.0 ± 16.9	0.574
BMI (kg m^−2^)	22.5 ± 4.6	21.8 ± 6.4	0.651
APACHE II	24.7 ± 6.6	23.9 ± 7.6	0.664
SOFA	8.5 ± 3.5	8.2 ± 3.9	0.653
Comorbidities			
CCI score	5.6 ± 2.3	4.6 ± 2.3	0.044
Chronic pulmonary disease	17 (60.7)	21 (61.8)	0.933
Chronic heart failure	12 (42.9)	9 (26.5)	0.175
Malignant disease	4 (14.3)	3 (8.8)	0.691
Diabetes	13 (46.4)	12 (35.3)	0.374
Septic shock	14 (50.0)	14 (41.2)	0.487
Baseline condition			
ALB (g L^−1^)	35.6 ± 5.0	35.1 ± 6.2	0.766
Baseline CL_CR_ (ml min^−1^)	76.4 ± 30.0	100.9 ± 24.4	0.001
CL_CR_ < 60 ml min^−1^	12 (42.9)	1 (2.9)	0.000
Polymyxin B dose regimen			
Load at first dose	23 (82.1)	26 (76.6)	0.585
Daily dose (mg kg day^−1^)	1.7 ± 0.3	1.8 ± 0.5	0.154
Daily dose ≥ 150 mg day^−1^	2 (7.1)	5 (14.7)	0.442
Cumulative dose (mg)	1175 (775–1837.5)	975 (550–1650)	0.190
Treatment time (days)	11 (7–18)	9 (5–17)	0.236
TDM	16 (57.1)	19 (55.9)	0.961
AUC_SS,24h_ 50–100 mg h L^−1^	11 (39.3)	8 (23.5)	0.180
AUC_SS,24h_ > 100 mg h L^−1^	2 (7.1)	4 (11.8)	0.681
Concomitant drugs			
Vancomycin	11 (39.3)	10 (29.4)	0.414
Tigecycline	14 (50.0)	14 (41.2)	0.487
Aminoglycoside	9 (32.1)	11 (32.4)	0.986
Norepinephrine	21 (75.0)	24 (70.6)	0.698
Loop diuretic	26 (92.9)	24 (70.6)	0.027
Positive fluid balance	14 (50.0)	20 (58.8)	0.487
CRRT	5 (17.9)	6 (17.6)	1.00
Mortality	13 (46.4)	17 (50.0)	0.779

*Note*: Data are presented as *n* (%), mean + SD, or median (IQR).

Abbreviations: ALB, albumin; APACHE, acute physiology and chronic health evaluation; AUC_SS,24h_, area under the concentration–time curve across 24 h at steady state; BMI, body mass index; CCI, Charlson comorbidity index; CL_CR_, creatinine clearance; CRRT, continuous renal replacement therapy; SOFA, sequential organ failure assessment; TDM, therapeutic drug monitoring.

### 3.1 Analysis of clinical response

**FIGURE 1:**
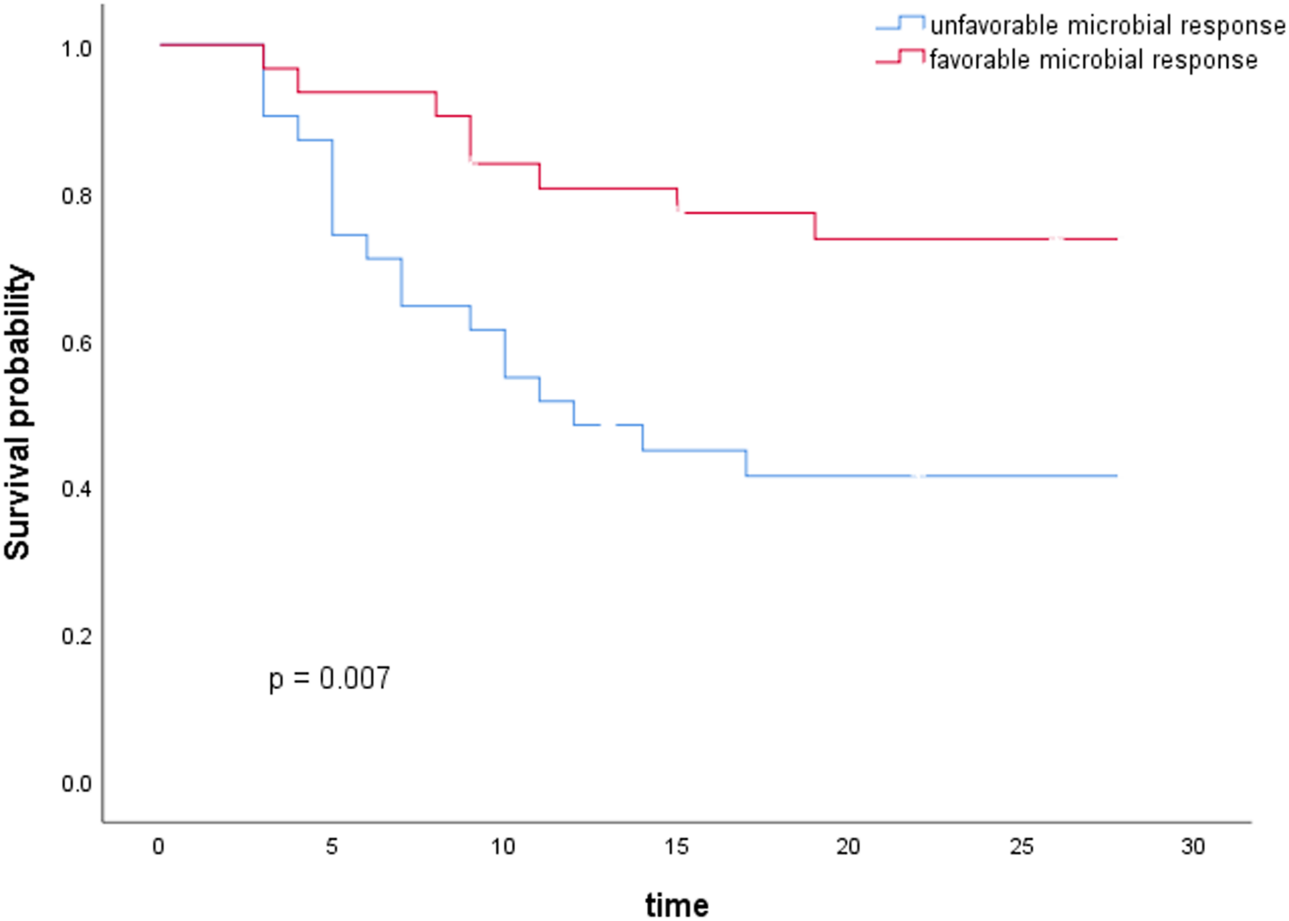
Kaplan–Meier estimates of survival and mortality during 28 days after polymyxin B treatment

We compared the distribution of various characteristics between the groups with favorable and unfavorable clinical response (Table 1). In the multivariate analysis, only three variables were independently associated with a favorable clinical response to polymyxin B therapy: complicated with septic shock (odds ratio [OR] = 0.12, 95% confidence interval [CI] 0.02–0.72; *p* = 0.021), reaching the target value of AUC_ss,24h_ (OR = 13.15, 95% CI 1.64–105.45; *p* = 0.015), and having a favorable microbiologic response (OR = 40.80, 95% CI 5.89–282.59; *p* = 0.00) (Table 3). Using Kaplan–Meier curves to compare survival probability of patients with favorable and unfavorable microbial response, there was a significant difference in the distribution of survival between the two groups (*p* = 0.007) (Figure 1).

**TABLE 3:** Multivariate analysis of clinical response to polymyxin B (*R* ^2^ = 0.675)

Variable	OR (95% CI)	*p* value
Female	2.85 (0.52–15.65)	0.229
Septic shock	0.12 (0.02–0.72)	0.021
Baseline PFR	1.01 (1.00–1.02)	0.132
Favorable microbiological efficacy	40.80 (5.89–282.59)	0.000
AUC_ss,24h_ reached the target value of 50–100 mg h L^−1^	13.15 (1.64–105.45)	0.015

Abbreviations: AUC_SS,24h_, area under the concentration–time curve across 24 h at steady state; CI, confidence interval; OR, odds ratio; PFR, PaO_2_/FiO_2_ ratio.

### 3.2 Analysis of nephrotoxicity

**FIGURE 2:**
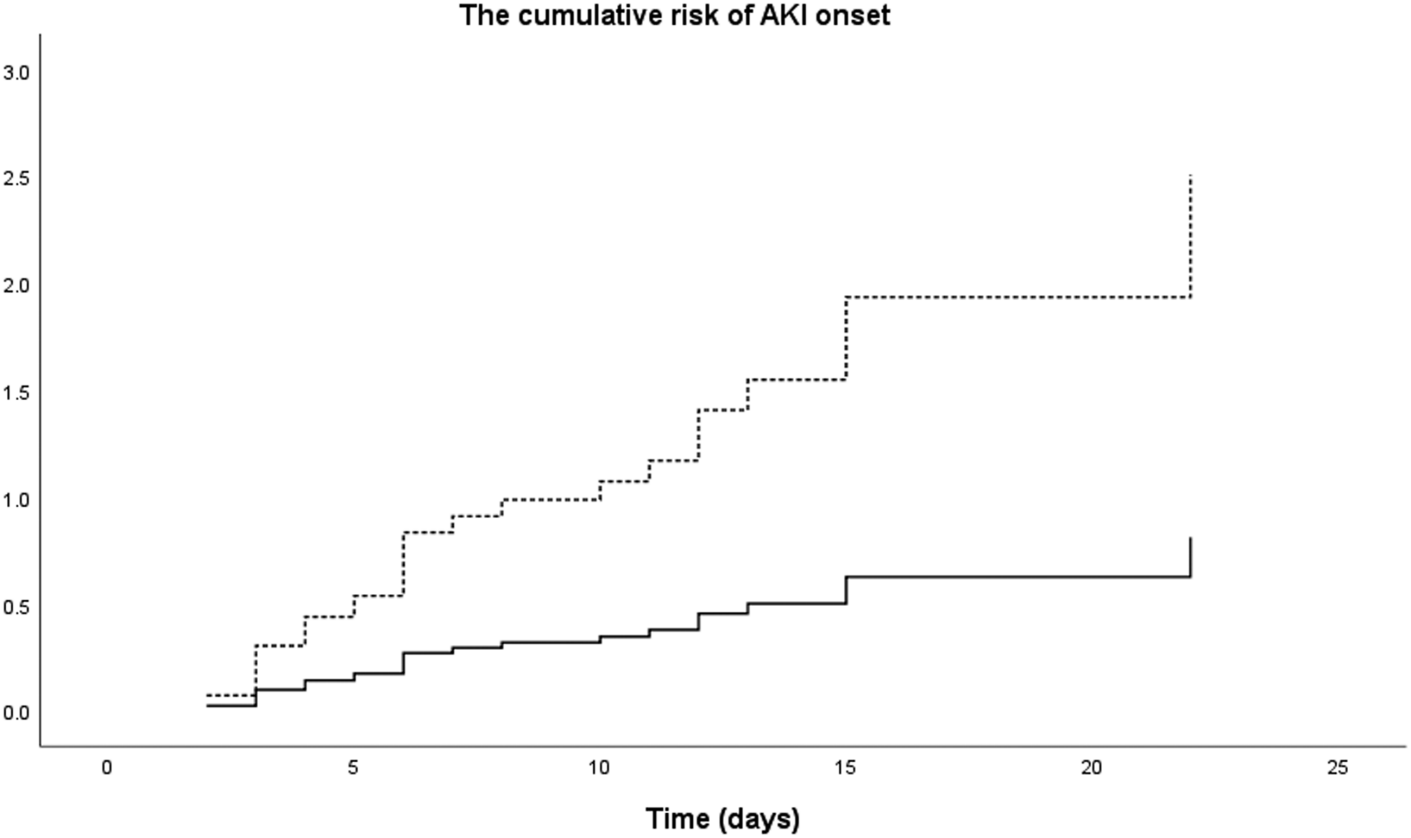
Comparison of nephrotoxicity developing. Compare the risk of AKI, *p* = 0.003; the broken line represents patients with CL_CR_ < 60 ml min^−1^, and the continuous line represents patients with CL_CR_ > 60 ml min^−1^. AKI, acute kidney injury

AKI occurred in 28 patients (45.2%) during polymyxin B therapy, of whom 15 (53.6%) were classified as risk, 9 (32.1%) as injury, and 4 (14.3%) as failure. The median time for the onset of AKI was 6 days (IQR 3.0–10.8). Clinical characteristics of patients with and without AKI were described in Table 2. In the multivariate analysis, baseline CL_CR_ (OR = 0.96, 95% CI 0.93–0.99; *p* = 0.008) and concomitant treatment with loop diuretics (OR = 5.93, 95% CI 1.03–34.07; *p* = 0.046) were independently associated with the onset of AKI (Table 4). Further Cox regression showed that patients with a CL_CR_ > 60 ml min^−1^ had a lower risk of AKI (OR = 0.323, 95% CI 0.153–0.685; *p* = 0.003) during polymyxin B therapy (Figure 2).

**TABLE 4:** Multivariate analysis of AKI (*R* ^2^ = 0.334)

Variable	OR (95% CI)	*p* value
CCI score	0.97 (0.71–1.32)	0.827
Loop diuretic	5.93 (1.03–34.07)	0.046
Baseline CL_CR_	0.96 (0.93–0.99)	0.008
Polymyxin B daily dose (mg kg day^−1^)	0.99 (0.94–1.04)	0.744

Abbreviations: AKI, acute kidney injury; CCI: Charlson comorbidity index; CI, confidence interval; CL_CR_, creatinine clearance; OR, odds ratio.

## 4 DISCUSSION

In this study, we identified the factors affecting the effectiveness and nephrotoxicity of polymyxin B in critically ill patients with nosocomial pneumonia and reported for the first time that the achievement of the optimal pharmacodynamic (PD) target of polymyxin B can improve the clinical treatment outcome. Our findings suggest that TDM of polymyxin B is a valuable intervention, which contributes to optimize the clinical application of polymyxin B in severe pulmonary infection.

Currently, the data for AUC_ss,24h_ target value for polymyxin B are lacking, and the guideline recommended a value similar to the target of colistin as an acceptable therapeutic target for polymyxin B.^10^ The evidence for this therapeutic target mainly come from in vitro experiments and meta‐analysis, which has not been evaluated within clinical setting in the targeted patient population. Our study reported for the first time that the achievement of this optimal PD goal was independently associated to favorable clinical effectiveness of polymyxin B in severe pneumonia. At present, polymyxin B is used as an antibiotic of last resort for patients with MDR and XDR Gram‐negative bacterial infections.^3^ Unfortunately, only a few studies have evaluated the factors that affect its efficacy.^7^, ^18^, ^19^ However, these studies did not measure the actual plasma exposure of polymyxin B, which is closely related to its antibacterial activity. The AUC_ss,24h_ is the main PD parameter to assess the plasma exposure of polymyxin B, and when the value of AUC_ss,24h_ is within the therapeutic target of 50–100 mg h L^−1^, polymyxin B can effectively eliminate polymyxin‐susceptible *A. baumannii*, *P. aeruginosa*, and *K. pneumoniae*, meantime its adverse effects are acceptable.^10^ Consistent with this finding, we can observe that a favorable microbiological efficacy was independently associated with a favorable clinical response, and HAP/VAP were more frequently cured in patients with reduced or eradicated causative pathogens. We used real‐world data to demonstrate the effect of reaching the target AUC_ss,24h_ value on improving treatment outcomes, and these findings are expected to help better define the optimal AUC_ss,24h_ value of polymyxin B and explore the relationship between plasma exposure and treatment success versus failure in severe pneumonia patients.

At present, TDM is widely used for patients treated with aminoglycosides or vancomycin, and its clinical advantage has been demonstrated in some studies.^12^ The extreme PK variability of antimicrobial agents in critically ill patients and the PK/PD characteristics of polymyxin B itself highlight the necessity of TDM in the course of treatment.^10^ However, to the best of our knowledge, there are no reports of introducing polymyxin B TDM into clinical practice. In our study, we performed TDM on most patients and found that reaching the therapeutic target of AUC_ss,24h_ was an independent predictor of treatment success. Our findings suggested that TDM is a valuable intervention that should be introduced more widely in clinical practice. It is necessary to conduct well‐designed controlled trials to evaluate the role of TDM in improving prognosis during polymyxin B treatment in the future.

Consistent with previous studies, complicated with septic shock was predictive of an unfavorable clinical response to polymyxin B therapy.^7^, ^20^, ^21^ Other factors, such as a high APACHE II score and CCI score, complicated with ARDS, were also reported to be independently associated with polymyxin B treatment failure in these studies. In our study, univariate analysis showed that patients with a lower baseline PFR had a poor response to polymyxin B treatment (151.9 [IQR 107.3–243.2] vs. 126.9 [IQR 84.8–160.6]; *p* = 0.049). These findings suggest that the severity of the patient's disease is directly related to the treatment outcome of polymyxin B. The analysis also showed that the treatment time in unfavorable response group was shorter (8.5 vs. 12.5, *p* = 0.064). Compared with the 7‐ to 8‐day course of antimicrobial therapy for HAP/VAP guidelines,^14^ the prolonged treatment time of favorable response group might be related to the severe complications and drug‐resistant bacterial infections of the patients included in our study. On the other hand, the shorter course of polymyxin B therapy in unfavorable group could not rule out the influence of early mortality of severe cases.

Compared with the polymyxin B dosage recommended by some consensus,^10^, ^14^ our daily dose is smaller, and we considered to be related to a number of reasons. First of all, according to the data of China Surveillance Network for Bacterial Resistance (CHINET) and Chinese Antimicrobial Resistance Surveillance of Nosocomial Infection (CARES),^22^ the main drug‐resistant Gram‐negative bacteria in HAP/VAP have a good sensitivity to polymyxin B. This is consistent with our data, which showed that most of the isolates cultured were highly susceptible to polymyxin (MIC ≤ 0.5 mg L^−1^). In this case, based on the results of the clinical PK study of polymyxin B in critically ill patients,^23^ our dose was appropriate, which is also the dose recommended in the product label (1.5–2.5 mg kg day^−1^). Given the poor correlation between the polymyxin B PK and CL_CR_,^10^ we did not adjust the daily maintenance dose according to the patient's renal function. Second, clinical PK/PD data that can be used to guide the use of polymyxin B in Chinese patients are very scarce, and more information are needed to balance efficacy and safety, which is the significance of our study. Finally, it is important to recognize that in Chinese mainland, only drug instructions and pharmacopeia have legal significance; the guidelines and consensus are only for clinical reference. In our study, although the polymyxin B dose was small, the proportion of AUC_ss,24h_ reaching the therapeutic target was acceptable.

The prevalence of AKI in our study was 45.1%, which was similar to the results of a recent meta‐analysis.^6^ This prior study showed that among 2994 patients (from 28 studies) who received intravenous polymyxin B, the all‐cause nephrotoxicity was 40.7%. John et al. retrospectively analyzed the safety of receiving high‐dose intravenous polymyxin B (3.61 ± 0.97 mg kg day^−1^),^24^ and the prevalence of AKI in their population was 47.0%, similar to our results, although the dose was higher than ours. These data indicate that the incidence of polymyxin B‐related nephrotoxicity may not increase with an increasing dose. Rigatto et al. also found that the occurrence of AKI no longer increased when the dose of polymyxin B exceeded a certain threshold (150 mg day^−1^ in their study).^25^ Therefore, they speculated that there might be a toxicity threshold. Animal studies have revealed that the pathogenesis of polymyxin B‐associated nephrotoxicity is related to drug reabsorption and deposition in proximal convoluted tubule cells in the kidney.^26^ Additionally, it has been shown that tubular reabsorption of polymyxin B seems to follow a saturable nonpassive mechanism.^27^ Our findings contribute to this body of work and will help to enhance the knowledge of the pathogenesis of polymyxin B‐related nephrotoxicity.

In our study, a high level of serum creatinine was an independent risk factor for polymyxin B‐related AKI. Cox regression analysis showed that patients with CL_CR_ < 60 ml min^−1^ had a significantly increased risk of developing AKI. A prospective multicenter study conducted by Rigatto et al. also reported similar findings.^25^ However, there are some research findings that are contrary to ours: John et al. found that a higher serum creatinine was a protective factor for the onset of AKI, and they speculated that this could be explained by the fact that low glomerular filtration reduced the reabsorption of polymyxin B in the renal tubules.^24^ However, as discussed above, their study enrolled patients receiving high‐dose intravenous polymyxin B, and the prevalence of AKI did not increase compared with other studies using conventional doses. This does not seem to be consistent with their explanation. Crass et al. found that higher level of serum creatinine might be protective against polymyxin nephrotoxicity in patients with cystic fibrosis.^28^ However, their research also suggested that cystic fibrosis itself was a protective factor, which might compromise the accuracy of their conclusions. At present, there is no consistent opinion on the association between baseline renal insufficiency and polymyxin B‐related nephrotoxicity. Our research shows that patients with underlying renal impairment may be more susceptible to developing further injury, and timely monitoring of renal function is critical to detect AKI occurrence.

Finally, we found that concomitant treatment with loop diuretics was independently associated with polymyxin B‐related nephrotoxicity. Although relevant risk factors vary between studies, some common factors have been identified throughout the literature,^29^ such as aminoglycosides, vancomycin, or amphotericin B, yet there are few reports on loop diuretics. Studies on vancomycin‐related nephrotoxicity have shown that the combined use of loop diuretics is a common risk factor.^30^ Although these studies did not report volume management in the included patients, the researchers speculated that the hypoperfusion status caused by diuretics increased the patient's risk of renal impairment. In our study, the patients were mainly in positive fluid balance during polymyxin B treatment, and there was no significant difference in fluid management between the AKI group and the non‐AKI group. Hypoperfusion did not seem to be sufficient to account for the role of diuretics in polymyxin B‐associated nephrotoxicity. The mechanism of diuretics increasing the risk of AKI during nephrotoxic antibiotic treatment needs to be further studied.

There are several limitations to our study. First, the sample size was small, and it may not be possible to test all potential factors affecting the efficacy and nephrotoxicity of polymyxin B. More samples should be enrolled to enhance the robustness of our results. Second, as a retrospective study, we could not perform TDM for all patients included in the study, resulting in unknown AUC_ss,24h_ values for some patients, and a prospective study is needed to verify our study results.

## 5 CONCLUSION

In conclusion, our results show that TDM of polymyxin B is a valuable intervention, and the achievement of its optimal PD target can improve the treatment outcome. In addition, the severity of the disease is a reliable risk factor for polymyxin B treatment failure. Finally, renal insufficiency and concomitant treatment with loop diuretics were found to be associated with the risk of nephrotoxicity. These findings provide important information for maximizing the clinical efficacy and minimizing nephrotoxicity.

## CONFLICT OF INTEREST

None of the authors have conflicting interests that interfere with the integrity of the content of the article.

## ETHICAL STATEMENT

This study was approved and supervised by the Ethics Committee of China‐Japan Friendship Hospital (NO.2019‐156‐K105), and written informed consent was waived.

## AUTHOR CONTRIBUTIONS

All authors made substantial contributions to the conception and design of the study or to the data acquisition, analysis, or interpretation; reviewed and approved the final manuscript; and significantly contributed to this study. Qingyuan Zhan took full responsibility for the integrity of the submission and publication and was involved in the study design. Qingyuan Zhan and Qinghua Ye participated in the design of the study and coordination. Qinghua Ye, Qianlin Wang, Ziying Chen, and Wenqian Chen involved in data collection, had full access to all of the data in the study, took responsibility for the integrity of the data, and were responsible for data verification. Qinghua Ye took the responsibility for statistical analysis and drafted the manuscript. Qianlin Wang provided crucial revision for important intellectual content. All authors read and approved the final manuscript.

## Data Availability

The data used to support the findings of this study are available from the corresponding author on reasonable request.
